# Social media as a source of medical information during COVID-19

**DOI:** 10.1080/10872981.2020.1791467

**Published:** 2020-07-09

**Authors:** Michael Samy, Rebecca Abdelmalak, Amna Ahmed, Mary Kelada

**Affiliations:** Imperial College School of Medicine, Faculty of Medicine, Imperial College London, London, UK ms4716@ic.ac.uk

Dear Editor,

The COVID-19 pandemic has been rapidly progressing, with guidelines and advice constantly evolving in light of emerging and updated evidence. This has warranted the need for rapid dissemination of information. The internet is becoming an increasingly popular source of medical information, especially at a time in which data can be incomplete and originate from different sources. Social media offers a platform where all individuals can freely access medical information; from evidence-based data produced by medical professionals to opinion-based information from a laypersons’ first-hand experiences of health and illness.

Guidance issued by the General Medical Council uses the term ‘social media’ to encompass blogs and microblogs (such as Twitter), internet forums (such as doctors.net), content communities (such as YouTube), and social networking sites (such as Facebook and LinkedIn) [1]. These have all been employed in attempts to spread information regarding the COVID-19 pandemic. For example, official government channels on Twitter and Instagram regularly published the latest government statistics and advice, whilst encouraging the public to follow guidelines. Slogans such as ‘Stay at Home, Protect the NHS, Save Lives’ were able to further their reach through social media. The government has also advertised on platforms frequented primarily by teenagers, such as TikTok, enabling information to reach audiences of all ages. This may be more difficult with traditional advertising methods. Hand hygiene is of extreme importance in the prevention of COVID-19 transmission [2], and YouTube videos were consequently created to demonstrate proper handwashing techniques to the public. YouTube videos were also utilised for the education of health professionals, such as to demonstrate proper donning of personal protective equipment. Clinicians’ forums, such as Facebook’s 15,000-member *COVID Doctors Forum (UK)*, have provided doctors with a community where they can reflect on emerging evidence and guidelines whilst offering support to fellow colleagues.

Practical issues arise surrounding the accuracy of information and reliability of those publishing content. Ethical concerns range from digital identity to proper professional behaviour in the use of social media, particularly confidentiality, defamation, and doctor-patient boundaries [3]. Furthermore, unverified medical anecdotes often evoke fear, while the desire to protect loved ones can quickly lead to hoax messages becoming viral. News outlets and social media platforms ought to be cautious about the information they disseminate. There are also concerns surrounding the distribution of advice by health professionals or people in authority, as illustrated by President Trump’s comments promoting the use of hydroxychloroquine for COVID-19 prevention without US Food and Drug Administration approval [4]. Moreover, the urgent need to disseminate information regarding COVID-19 may have led to some medical journals adopting an expedited peer-review process, perhaps compromising the quality of evidence published.

Social media plays a key role in making medical knowledge widely available. Information can be constantly updated and disseminated, which is vital in the early days of a rapidly evolving outbreak. However, the unregulated nature of the internet can result in unvalidated or unproven information being spread. This can lead to severe, and in some cases life-threatening, consequences. Therefore, the public need to be able to discern between sources that are trustworthy and unreliable. We would also argue that despite its challenges, social media platforms should play a greater role in the regulation and fact-checking of information distributed on their sites.
